# Impact of economic sanctions on child health in Cuba

**DOI:** 10.1136/bmjpo-2026-004510

**Published:** 2026-02-27

**Authors:** Angel Arturo Escobedo, Yaxsier de Armas, Paul Jonas, Imti Choonara

**Affiliations:** 1 Department of Epidemiology, Institute of Gastroenterology, Havana, Cuba; 2 Institute of Tropical Medicine “Pedro Kourí”, Havana, Cuba; 3 Faculty of Medicine, Leiden University, Leiden, Netherlands; 4 University of Nottingham School of Medicine, Derby, UK

**Keywords:** Child Health, Health Policy

 Child health in Cuba has been a major success of the Cuban revolution. Child mortality has been lower than in neighbouring countries and education has been universal and free. More recently, however, child mortality rates have been increasing. Under-5 child mortality rates have increased from 6.0 to 8.3 per 1000 live births over the last decade. Infant mortality has also increased from 4.0 to 9.9 per 1000 live births.^1^ One needs to understand why this has been happening.

The USA has imposed economic sanctions on Cuba since 1962—the longest lasting sanctions on any country. The United Nations (UN) General Assembly and the UN Human Rights Council have repeatedly called for the lifting of the sanctions by the American Government on Cuba. The USA imposes sanctions on more countries than the UN Security Council or any other country.^2^ It currently has sanctions on 16 countries.^3^ As the main global superpower, the impact of economic sanctions is considerable. Sanctions by the USA have a major impact on mortality in both children and adults and are estimated to result in >500 000 deaths worldwide annually.^4^

The impact of the US sanctions on Cuba has been highlighted in a report by the UN Special Rapporteur.^5^ It highlights the designation of Cuba as a state sponsor of terrorism in January 2021 by the US Government and the applying of extraterritorial jurisdiction to non-US companies. This has resulted in fines for international banks by the US Government (US$3 million for the Swiss bank EFG International in 2024).^5^ This has resulted in 200 foreign banks and financial institutions stopping dealing with Cuba since 2021.^5^ These changes have made foreign companies reluctant to invest in Cuba and made it more difficult for Cuba to trade with other countries. This has resulted in major economic difficulties for Cuba. UN institutions, such as UNESCO have been reluctant to help Cuba, as they have been worried that the USA will then withdraw funding.^5^

US sanctions prohibit the selling of technology that contains 10%+ of US components to Cuba.^6^ This means that medical equipment ranging from infusion pumps, cardiac catheters, haemodialysis machines and ventilators that are produced by US companies or non-US companies that contain 10%+ US components cannot be sold to Cuba. This has resulted in shortages of medicines, testing reagents, equipment and consumables within the healthcare system. The US sanctions do not technically prohibit the sale of medicines to Cuba. Pharmaceutical companies can apply to the US Government for a license to sell medicines to Cuba. In practice, however, licenses are rarely issued.^6^

The effect of the US sanctions on Cuba is detailed in the report by Cuba to the UN.^7^ The National List of Essential Medicines in Cuba includes 651 items.^7^ 250 medicines are imported, and the remaining 401 are manufactured in Cuba. Over 50% of the medicines (364 medicines) are in short supply. This includes medicines manufactured in Cuba, where the reagents and ingredients are not readily available from outside the USA. This results in difficulties for parents of children with chronic illnesses, such as diabetes or asthma, with the uncertainty in them receiving their regular treatment, which can lead to complications. Similarly, children with acute illnesses may be denied treatment at home due to the unavailability of certain medicines. The US Government is now banning Cuban scientists from accessing scientific information on 21 biomedical databases of the National Institutes of Health.^7^

The leading supplier of dialysis machines in Latin America is Baxter Healthcare, an American company, and refuses to sell equipment to Cuba.^7^ Continuous insulin infusion pumps for children with type 1 diabetes, endoscopic surgical equipment and diagnostic reagents and equipment are all affected. Some chemotherapeutic medicines used for childhood malignancies are unavailable, resulting in Cuban doctors having to modify established treatment regimes. This has resulted in survival rates falling from 75% to 60%.^6^ Surgery has been affected, due to shortages in medicines, consumables and equipment. There are 9913 children awaiting surgery because of shortages.^7^

Cuba has carried out regular fumigation of houses and flats to reduce the risk of dengue and other mosquito-transmitted diseases. However, due to fuel shortages, fumigation has been carried out less frequently. In 2025, Cuba has experienced outbreaks of dengue, Oropouche and chikungunya. These arboviruses are endemic in Central and South America, but have been exacerbated by sanitation issues in Cuba. It has been estimated that one-third of the population has experienced an arbovirus infection.^8 9^

Additionally, there are now shortages in the critical infrastructure of power plants leading to frequent blackouts, water supply and sanitation alongside food production and storage. Electricity blackouts have an adverse effect on water sanitation. This has resulted in the migration of young people, leading to vacancies in healthcare for the first time. This directly affects the quality of care that children receive. Alongside causing problems in public services, the migration of young educated professionals has a major financial impact on Cuba economically, as education including university is free in Cuba, that is, the government pays for it. The migration also affects families, in that one parent may migrate abroad, leaving the other parent to look after the children as a single parent. This may result in both financial difficulties for the family and psychological problems for the children.

Food shortages are a significant problem for infants, young children and pregnant women. Growth and development are adversely affected by food shortages, and malnutrition weakens the immune system, resulting in an increased vulnerability to infections. Food production alongside its processing, conservation and transport has fallen due to a combination of economic problems, difficulties in importing products and fuel shortages.^5^ The import of powdered milk, flour, poultry, beef and pork has fallen dramatically since the designation of Cuba as a state sponsor of terrorism in 2021.^5^

Despite the additional (>200) sanctions measures and its designation as a state sponsor of terrorism by the USA in 2021, Cuba has managed to keep its healthcare system functioning and free of charge to all.^6^ The impact of the economic sanctions on Cuba by the USA is considered successful by the US Government. The deliberate destruction of a healthcare system in a small middle-income country by a neighbouring superpower, however, should be considered unacceptable by all health professionals. The latest US executive order declaring Cuba a threat to the security of the USA and imposing tariffs on any country that supplies oil to Cuba is equivalent to imposing a siege on a country.^10^ No healthcare system can survive without electricity, and Cuba is still dependent on oil for electricity production.

During warfare, it is unacceptable to target innocent civilians. There are, however, no controls on the impact of sanctions, which directly impact on the most vulnerable. International institutions, such as the UN and the Red Cross, need to prevent unilateral sanctions destroying healthcare.

## References

[1] Cuban Ministry of Health. https://salud.msp.gob.cu/?p=45568&doing_wp_cron=1768232345.3217940330505371093750.

[2] United Nations security council. https://main.un.org/securitycouncil/en/sanctions/information.

[3] US Dept of State Economic sanctions programs. https://www.state.gov/division-for-counter-threat-finance-and-sanctions/economic-sanctions-programs/page/2/.

[4] Rodríguez F, Rendón S, Weisbrot M (2025). Effects of international sanctions on age-specific mortality: a cross-national panel data analysis. Lancet Glob Health.

[5] United Nations human rights council. https://www.ohchr.org/sites/default/files/statements/20251121-eom-stm-cuba-sr-negative-impact-unilateral-en.pdf.

[6] US Department of State Cuba sanctions. https://www.state.gov/cuba-sanctions/.

[7] CUBA’S REPORT (2025). Pursuant to United Nations General Assembly Resolution 79/7, entitled “Necessity of ending the economic, commercial, and financial blockade imposed by the United States of America against Cuba”. https://cubaminrex.cu/sites/default/files/2025-09/InformeB2025i_1.pdf.

[8] Beacon (2025). Cuba reports 47 003 chikungunya cases and 52 arboviral deaths; pediatric population most affected. https://beaconbio.org/en/report/?reportid=c57efc8c-99ef-47dc-9e02-5e97cc94bb76&eventid=6107038b-5f42-4a39-ae59-95619ca03b9d.

[9] MINSAP confirma un total de 52 fallecidos por chikungunya y dengue en Cuba y reporta descenso gradual de casos. https://www.directoriocubano.info/acontecer/minsap-confirma-un-total-de-52-fallecidos-por-chikungunya-y-dengue-y-reporta-descenso-gradual-de-casos/.

[10] White House Addressing threats to the US by the Government of Cuba. https://www.whitehouse.gov/presidential-actions/2026/01/addressing-threats-to-the-united-states-by-the-government-of-cuba/.

