# Supplementary material for: Impact of economic sanctions on child health in Cuba
Source: BMJ Paediatr Open. 2026 Feb 27;10(1):e004510. doi: 10.1136/bmjpo-2026-004510 (PMC12959023; doi:10.1136/bmjpo-2026-004510)
Supplement: Supplementary file 1 [file bmjpo-2026-004510.reviewer_comments.pdf]

PEER REVIEW HISTORY

BMJ Paediatrics Open publishes all reviews undertaken for accepted manuscripts. Reviewers are asked to complete a checklist review form and are provided with free text boxes to elaborate on their assessment. These free text comments are reproduced below.

ARTICLE DETAILS

**TITLE (PROVISIONAL)**	Impact of economic sanctions on child health in Cuba
**AUTHORS**	Escobedo, Angel Arturo<br>de Armas, Yaxsier<br>Jonas, Paul<br>Choonara, Imti

VERSION 1 – REVIEW

**REVIEWER NAME**	Gunnlaugsson, Geir
**REVIEWER AFFILIATION**	Faculty of Social and Human Sciences, University of Iceland
**REVIEWER CONFLICT OF INTEREST**	No Conflict of Interest
**AI DISCLOSURE**	No, I have not used AI
**DATE REVIEW RETURNED**	27-Jan-2026

**GENERAL COMMENTS**	The Viewpoint is timely and powerful, calling attention to the impact economic sanctions can have on child health with Cuba as a case in point. It is overall well written and makes a strong case for lifting economic sanctions that target healthcare, such as those of the USA on Cuba.<br>I have suggestions for minor revisions for the authors to consider.<br>• Page 2, line 27, the term USA is defined. Later (line 40), the term US is also defined. Suggest sticking to one definition and using it consistently.<br>• Page 2, line 30, states that the UN has never imposed sanctions on Cuba. It follows the discussion of sanctions by the Security Council, which is confusing, as the Security Council is part of the UN. Suggest deleting this sentence or making a better point about the fact that the UN refers to the General Assembly.<br>• Page 2, line 33, highlights that the US has economic sanctions on at least 16 countries. Suggest using the exact number from the list.<br>• Page 2, line 34, the International Criminal Court is mentioned. Suggest either deleting the sentence or moving it elsewhere, as it interrupts the flow of reading with this additional (important) detail.<br>• On page 2, lines 40–41, the word 'report' is mentioned twice.<br>• Page 2, line 44, EFG International is mentioned. For readers who may not know this is a Swiss bank, the term needs minor clarification.<br>• Page 2, line 46: UNESCO is identified as one of the UN financial institutions, rather than as one focused on education. Needs clarification.<br>• Page 3, lines 31–32, one sentence, one paragraph. Suggest integrating elsewhere in the text.<br>• On page 3, line 45, there is a paragraph on food shortages and growth. It is short and might fit better elsewhere, e.g., in the first paragraph on page 2.<br>• Page 3, lines 34–35, make clear that the electric blackouts negatively impact water supply and sanitation, etc.

	• Citations are either within a sentence, followed by a punctum, or after punctum. Needs to be formatted in line with the BMJ PO standard.<br>• Correct minor errors in text, such as lacking space (e.g., page 2, line 37 and page 3, line 40).

**REVIEWER NAME**	Spencer, Nick
**REVIEWER AFFILIATION**	University of Warwick Warwick Medical School, Division of Mental Health and Wellbeing
**REVIEWER CONFLICT OF INTEREST**	I have published with Imti Choonara
**AI DISCLOSURE**	No, I have not used AI
**DATE REVIEW RETURNED**	05-Feb-2026

**GENERAL COMMENTS**	Since January 2025, major concern has been expressed about the impact on infants and children in low and middle income countries of the Trump administration's decision to cut the USAID budget potentially leading to millions of infant and early childhood deaths due to removal of funding for essential medical services and supplies (see https://doi.org/10.1136/bmj.r600). This viewpoint shows that US administrations, since the early 1960s, have been imposing economic sanctions on Cuba that have had similar adverse effects on infant and child health as the recent global USAID cuts. The authors detail the effects of sanctions on many imports that are essential to sustaining the health and well-being of the population especially the most vulnerable such as pregnant women, infants, children and the elderly.<br>The authors point out that, despite the sanctions, Cuba has managed to sustain health and education services that are fully government funded and free at time of use but the sanctions have had adverse consequences for public health including a sharp deterioration of U5MR/IMR in last decade. However, while the paper outlines many of the specific sanctioned items that have limited the availability and effectiveness of treatment for conditions such as diabetes, it does not attempt to explain why, when sanctions have been in place for more than 60 years, the deterioration in mortality rates has been so sharp in the last decade. Is it specific sanctions introduced in last decade or cumulative effects of the extraordinarily long period of continuous sanctions? I think these questions are important to understanding both the direct effects of sanctions and how the resilience of Cuban economy has been undermined.<br>To inform an explanation for the deterioration in U5MR & IMR in the last 10 years, a breakdown of causes of death in these age groups might be useful. The authors might consider commenting on whether the causes throw further light on how the sanctions have impacted these deaths if the data are available and reliable.<br>Minor issues:<br>1. Funding – there is no funding declaration<br>2. References:<br>- Ref. 8 – I'm not familiar with citing videos but I think the source of the Belly of the Beast video needs to be detailed especially if it is a Cuban government site<br>- Ref. 9 – English translation needed<br>In summary, this is an important viewpoint demonstrating how sanctions directly affect infants and children who are among the most vulnerable in low and middle income countries. I've suggested the authors address in more detail reasons for deterioration in last 10 years – I think this will strengthen the paper

VERSION 1 – AUTHOR RESPONSE

Reviewer: 1
Prof. Geir Gunnlaugsson
Comments to the Author
The Viewpoint is timely and powerful, calling attention to the impact economic sanctions can have on child health with Cuba as a case in point. It is overall well written and makes a strong case for lifting economic sanctions that target healthcare, such as those of the USA on Cuba. THANK YOU
I have suggestions for minor revisions for the authors to consider.

- Page 2, line 27, the term USA is defined. Later (line 40), the term US is also defined. Suggest sticking to one definition and using it consistently. DONE
- Page 2, line 30, states that the UN has never imposed sanctions on Cuba. It follows the discussion of sanctions by the Security Council, which is confusing, as the Security Council is part of the UN. Suggest deleting this sentence or making a better point about the fact that the UN refers to the General Assembly. DELETED
- Page 2, line 33, highlights that the US has economic sanctions on at least 16 countries. Suggest using the exact number from the list. DONE 16 COUNTRIES STATED
- Page 2, line 34, the International Criminal Court is mentioned. Suggest either deleting the sentence or moving it elsewhere, as it interrupts the flow of reading with this additional (important) detail. DELETED
- On page 2, lines 40–41, the word ‘report’ is mentioned twice. DELETED
- Page 2, line 44, EFG International is mentioned. For readers who may not know this is a Swiss bank, the term needs minor clarification. CLARIFIED
- Page 2, line 46: UNESCO is identified as one of the UN financial institutions, rather than as one focused on education. Needs clarification. FINANCIAL DELETED
- Page 3, lines 31–32, one sentence, one paragraph. Suggest integrating elsewhere in the text. MOVED TO END OF PARA 5
- On page 3, line 45, there is a paragraph on food shortages and growth. It is short and might fit better elsewhere, e.g., in the first paragraph on page 2. PARA EXPANDED
- Page 3, lines 34–35, make clear that the electric blackouts negatively impact water supply and sanitation, etc. DONE
- Citations are either within a sentence, followed by a punctum, or after punctum. Needs to be formatted in line with the BMJ PO standard. THANK YOU CORRECTED
- Correct minor errors in text, such as lacking space (e.g., page 2, line 37 and page 3, line 40). THANK YOU CORRECTED

Reviewer: 2
Dr. Nick Spencer, University of Warwick Warwick Medical School
Comments to the Author
Since January 2025, major concern has been expressed about the impact on infants and children in low and middle income countries of the Trump administration’s decision to cut the USAID budget potentially leading to millions of infant and early childhood deaths due to removal of funding for essential medical services and supplies (see https://doi.org/10.1136/bmj.r600). This viewpoint shows that US administrations, since the early 1960s, have been imposing economic sanctions on Cuba that have had similar adverse effects on infant and child health as the recent global USAID cuts. The authors detail the effects of sanctions on many imports that are essential to sustaining the health and well-being of the population especially the most vulnerable such as pregnant women, infants, children and the elderly.
The authors point out that, despite the sanctions, Cuba has managed to sustain health and education services that are fully government funded and free at time of use but the sanctions have had adverse consequences for public health including a sharp deterioration of U5MR/IMR in last decade. However,

while the paper outlines many of the specific sanctioned items that have limited the availability and effectiveness of treatment for conditions such as diabetes, it does not attempt to explain why, when sanctions have been in place for more than 60 years, the deterioration in mortality rates has been so sharp in the last decade. Is it specific sanctions introduced in last decade or cumulative effects of the extraordinarily long period of continuous sanctions? I think these questions are important to understanding both the direct effects of sanctions and how the resilience of Cuban economy has been undermined. THANK YOU PARA 3 AND PENULTIMATE PARA EXPANDED EXPLAINING THE IMPACT OF DESIGNATION OF CUBA AS A STATE SPONSOR OF TERORISM

To inform an explanation for the deterioration in U5MR & IMR in the last 10 years, a breakdown of causes of death in these age groups might be useful. The authors might consider commenting on whether the causes throw further light on how the sanctions have impacted these deaths if the data are available and reliable. DATA NOT AVAILABLE

Minor issues:

1. Funding – there is no funding declaration
2. References:

- Ref. 8 – I'm not familiar with citing videos but I think the source of the Belly of the Beast video needs to be detailed especially if it is a Cuban government site REF 8 REPLACED
- Ref. 9 – English translation needed REF 9 REPLACED

REFERENCES 8 AND 9 ARE IN ENGLISH AND SPANISH RESPECTIVELY. THEY CONTAIN SIMILAR DATA

In summary, this is an important viewpoint demonstrating how sanctions directly affect infants and children who are among the most vulnerable in low and middle income countries. I've suggested the authors address in more detail reasons for deterioration in last 10 years – I think this will strengthen the paper
